# Clinical profile and diagnostic challenges of tuberculous meningitis among HIV infected patients - a retrospective study

**DOI:** 10.1186/1471-2334-14-S3-P16

**Published:** 2014-05-27

**Authors:** Sudheer Aree Parambil, Mamatha Thamme Gowda, Rajendra Prasad Sivaswamy

**Affiliations:** 1 Vivekananda Memorial Hospital, Sargur, Mysore, Karnataka, India; 2 JSS Medical College and Hospital, JSS University, Mysore, Karnataka, India

## Background

Tuberculosis is the most common opportunistic infection among people living with HIV in India. Tuberculosis meningitis is the most serious form of TB with high morbidity and mortality, with diagnostic challenges in resource poor settings. TB meningitis (TBM) presents atypically especially when CD4 is low with fewer signs of meningeal irritation and non specific CSF picture. Widespread availability of ART has changed the clinical presentation of TBM with more patients presenting as unmasking TB Immune Reconstitution Inflammatory Syndrome (IRIS). In this study we propose to study the clinical profile of HIV infected patients with presumptive diagnosis of TB meningitis and to assess the utility of a diagnostic criteria for diagnosis of TB meningitis validated in South Africa.

## Methods

Retrospective descriptive study conducted among 100 consecutive HIV infected patients presumptively diagnosed to have TB meningitis admitted at a secondary care center.

## Results

Fever was the only symptom in 20% of patients. 89% of the patients had elevated CSF protein with only 7% having other CSF features suggestive of TBM. Fifty percentage of the patients had TB IRIS, among them 39.19% were unmasking type, and 10% were of paradoxical type of IRIS. In patients with stage I TBM only 35% fulfilled proposed diagnostic criteria, whilst in stage II and III 65% and 66%, respectively fulfilled diagnostic criteria.

## Conclusion

The study shows the proposed diagnostic criteria is not useful in early stages of TBM which was statistically significant. The utility of proposed diagnostic criteria was uniform among patients irrespective of duration of ART.

